# Charge transport in semiconductors assembled from nanocrystal quantum dots

**DOI:** 10.1038/s41467-020-16560-7

**Published:** 2020-06-05

**Authors:** Nuri Yazdani, Samuel Andermatt, Maksym Yarema, Vasco Farto, Mohammad Hossein Bani-Hashemian, Sebastian Volk, Weyde M. M. Lin, Olesya Yarema, Mathieu Luisier, Vanessa Wood

**Affiliations:** 1 Materials and Device Engineering Group, Department of Information Technology and Electrical Engineering, ETH Zurich, 8092 Zurich, Switzerland; 2 Nano TCAD Group, Department of Information Technology and Electrical Engineering, ETH Zurich, 8092 Zurich, Switzerland

**Keywords:** Electronic properties and materials, Quantum dots

## Abstract

The potential of semiconductors assembled from nanocrystals has been demonstrated for a broad array of electronic and optoelectronic devices, including transistors, light emitting diodes, solar cells, photodetectors, thermoelectrics, and phase change memory cells. Despite the commercial success of nanocrystal quantum dots as optical absorbers and emitters, applications involving charge transport through nanocrystal semiconductors have eluded exploitation due to the inability to predictively control their electronic properties. Here, we perform large-scale, ab initio simulations to understand carrier transport, generation, and trapping in strongly confined nanocrystal quantum dot-based semiconductors from first principles. We use these findings to build a predictive model for charge transport in these materials, which we validate experimentally. Our insights provide a path for systematic engineering of these semiconductors, which in fact offer previously unexplored opportunities for tunability not achievable in other semiconductor systems.

## Abstract (web summary)

While efficiency of nanocrystal-based devices has improved, charge transport within semiconductors assembled from nanocrystal quantum dots has remained unclear. Here, the authors use ab initio calculations to develop a predictive model for charge transport that also explains the origin of deep electronic traps and validate it experimentally.

## Introduction

Assembly of colloidal nanocrystal quantum dots (hereafter referred to simply as nanocrystals, NCs) into thin films^1^ is envisaged as a means to achieve next-generation, solution-processed semiconductors with electronic properties (e.g., band gaps^2^, band-edge positions^3^, mobilities^4^, and free carrier densities^5^) that can be defined to match specific application requirements^6–10^. This tunability is enabled by a multi-dimensional design space, where size, shape, composition, surface termination, and packing of the NCs can be systematically and independently controlled. While parametric studies have demonstrated some of the scaling relations in this design space^11–15^, the fundamental mechanism driving charge transport in NC-based semiconductors has remained unclear, making it difficult to build up predictive models for charge transport in NC semiconductors or tap the full potential of NCs as building blocks for electronic materials through theory-guided design.

Here, we perform large-scale density functional theory (DFT)-based ab initio calculations and simulations to understand carrier transport, generation, and trapping in NC-based semiconductors. We use these findings to build and experimentally validate a predictive model for charge transport in these systems. This predictive model allows us to design NC semiconductors with unique properties not achievable in the bulk, and the fundamental insights into charge carrier dynamics sets a clear agenda for the development of NC chemistry and self-assembly to realize novel semiconductors.

## Results

### Polaron formation and reorganization energy

**Fig. 1:**
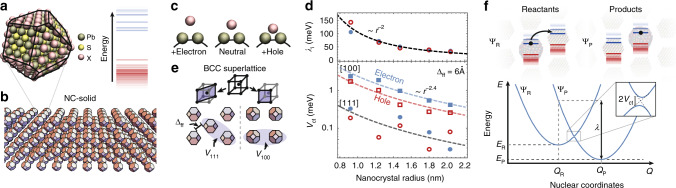
Charge transfer in NC solids. **a** Atomistic model of a PbS nanocrystal (NC) with halide (I) surface passivation and its quantized electronic structure. **b** Depiction of a thin film of nanocrystals (i.e, an NC solid). **c** Schematic of the nuclear reorganization, where the Pb–X bonds on the surface of the NC expand or shrink in the presence of an electron or hole. **d** Calculated reorganization energy (top) and electronic coupling (bottom) for electrons (blue) or holes (red) between [100] (squares) and [111] (circles) nearest neighbors (as depicted in **e**), assuming a facet-to-facet distance, Δ_ff_, of 6 Å in the [111] direction. **f** Configurational diagram for charge transfer between two nanocrystals, where an electron (black dot) moves from the nanocrystal on the left (configuration of reactants, *Q*_R_, with ground state energy *E*_R_) to the nanocrystal on the right (*Q*_P_ and energy *E*_R_). The reorganization energy (*λ*) and electronic coupling (*V*_ct_) is shown graphically.

Only recently has it been computationally feasible to treat the full atomic complexity of an NC ab initio, and this has proven key to understanding the mechanisms driving charge carrier dynamics on individual NCs^16^. In order to elucidate the mechanisms for charge transport in NC-based semiconductors, we implement large-scale DFT calculations on individual NCs, and on systems containing up to 125 NCs (Fig. 1a, b).

As a model system, we use lead sulfide (PbS) NCs in the strongly quantum confined regime (i.e., with radii *r* < *~3* nm) terminated with iodine ligands^17^. Since NC size is a parameter that is easy to systematically control in experiments, we perform calculations for different sized NCs in order to validate the resulting charge transport model with experiments. Details are provided in the Methods.

Before we understand how charge moves across an NC-based semiconductor, we must first consider the impact of the presence of a charge carrier on an individual NC. To do so, we compute the ground state physical structure of the NCs in their neutral charge state and when charged. Upon charging with an electron (or hole), the Pb–iodine ligand bonds on the (111) surfaces of the NCs expand (or contract), while the Pb–S bond lengths remain unchanged (Fig. 1c, Supplementary Fig. 1, Supplementary Information Note 1). Thus, the presence of a charge carrier on an NC can lead to the formation of a polaron. Because such polarons result from electrostatic interaction of the charge carrier with the negatively charged functional group of the ligands, polaron formation can be expected in any NC system with X-type ligands^18^ (e.g. halides, thiols, carboxylates).

Charge transfer from one NC to another thus implies a rearrangement of atoms at the surface of the two NCs. Although the shifts in bond length are small (up to 0.005 Å or 0.2% of the 3.22 Å nominal bond length), the associated reorganization energy for charge transfer between two NCs, *λ*, is large (10s to 100s of meV) (Fig. 1d). The reorganization energy decreases with increasing NC size due to a reduced carrier density across the NC and an increased number of ligands. In Supplementary Information Note 2, we discuss why it is reasonable to ignore the contribution to *λ* stemming from reorganization of the neighboring NCs (i.e., outer-shell reorganization).

### Electronic coupling in NC semiconductors

Having now understood that the presence of a charge carrier on an NC can lead to the formation of a polaron, we can determine the type of transport (band like or hopping) by calculating the electronic coupling between neighboring NCs, *V*_ct_.

Small-angle X-ray scattering measurements on PbS NCs have demonstrated that they assemble into a body-centered-cubic (BCC), face-center-cubic, and related structures, along with alignment of the individual NCs with respect to the superlattice structure as depicted in Fig. 1e for a BCC structure^19–21^. We consider two relative orientations for neighboring NCs: [111]-neighbors and [100]-neighbors (Fig. 1e). We calculate electron and hole couplings for both orientations (i.e., *V*_111_ and *V*_100_) over a range of *r* and inter-NC facet-to-facet distances, Δ_ff_ (Supplementary Fig. 2, Supplementary Information Note 3). The results for Δ_ff_ = 6 Å are shown in Fig. 1d. *V*_ct_ increases strongly as the size of the NC decreases in agreement with analytical calculations modeling the NCs as spherical potential wells^22^. This trend is explained by an increased carrier density on the outer atoms of the NC with increasing confinement in smaller NCs. The coupling in the [100] direction is about an order of magnitude larger than in the [111] direction, due to strong confinement of the carriers away from the ligand-rich [111] facets^16^.

### Phonon-assisted charge transfer

The fact that *V*_ct_ is more than an order of magnitude smaller than *λ* over the range of *r* and Δ_ff_ of typical PbS NC semiconductor with X-type ligands informs us that, in these systems, the charge carriers are polarons localized to individual NCs, and that charge transport occurs through a phonon-assisted charge transfer (polaron hopping) between neighboring NCs.

Since the charge carrier deforms the Pb-ligand bonds upon polaron formation, charge transfer will be driven by the Pb-ligand vibrations. Ab initio calculations of the phonon density-of-states of PbS NCs^16^, backed by inelastic neutron^23^ and X-ray^24^ scattering, indicate that Pb-ligand vibrations for common X-type ligands occur at energies *ћω* < ~15 meV. Therefore, at temperatures above ~175 K, charge transfer will occur at a rate^25^:

1 $$k_{{\mathrm{ct}}} = N_{\mathrm{P}}\frac{{2{\uppi}}}{\hbar }V_{{\mathrm{ct}}}^2\sqrt {\frac{1}{{4{\uppi}\lambda k_{\mathrm{B}}T}}} {\mathrm{e}}^{ - \left( {{\mathrm{\Delta }}E + \lambda } \right)^2/4\lambda k_{\mathrm{B}}T},$$

where Δ*E* = *E*_P_ − *E*_R_ (i.e. the energy of the products minus the energy of the reactants) and *N*_P_ is the number of degenerate product states (Fig. 1f). At temperatures below ~175 K, transfer rates will saturate to their temperature-independent, low-temperature limit (Supplementary Information Note 4), or, in the presence of disorder, transport will transition to an Efros–Shlovskii variable range hopping regime^26^.

Assuming an NC semiconductor of isoenergetic NCs and no applied field (Δ*E* = 0), Eq. (1) predicts charge transfer times on the order of 10–100s ps for PbS NC semiconductors at room temperature, in agreement with recent measurements^13^. Since intra-band carrier cooling rates in PbS NCs proceed at 100s fs times scales^27^, the reactant and product states are thus the highest occupied electronic states (in the case of hole transport) or the lowest unoccupied electronic states (in the case of electron transport). This is in agreement with experimental measurement of the mobility band gap in an NC semiconductor scaling linear with the band gap of the individual NCs^28^.

### Energetic landscapes in NC semiconductors

In a realistic NC semiconductor, Δ*E* ≠ 0, with differences in the alignment between the highest occupied (or lowest unoccupied) states of neighboring NCs contributing to Δ*E*. Since a large Δ*E* will have significant impact on the time scales of transport, it is therefore critical to understand and control the energetic landscape within an NC solid. One contribution to Δ*E* is the distribution of the individual NC band gaps, stemming from size and shape disorder of the constituent NCs. Additionally, deep, electronic trap states are known to exist in NC semiconductors^29^, and possible explanations of their origin include mid-gap states on individual NCs^30^ and fused NC dimers^31^. Here, we demonstrate oxidized or reduced doped NCs in the NC semiconductor also form electronic traps in NC semiconductors.

An individual NC is doped according to the oxidation-number sum rule^32,33^:

2 $$N_{\mathrm{C}}V_{\mathrm{C}} + N_{\mathrm{A}}V_{\mathrm{A}} + \mathop {\sum }\limits_i V_i = \left\{ {\begin{array}{*{20}{c}} {0,} & {{\mathrm{intrinsic}},} \\ { < 0,} & {{{p}} {\mbox{-}} {\mathrm{doped}},} \\ { > 0,} & {{{n}} {\mbox {-}} {\mathrm{doped}},} \end{array}} \right.$$

where *N*_x_ is the number of the cations (C) and anions (A), and *I* are the impurities and ligands with oxidation state *V*_*x*_ comprising the NC (e.g. for PbS NCs with halide ligands *V*_Pb_ = +2, *V*_S_ = −2, and *V*_l_ = −1). Doped NCs will in general be energetically unfavorable; however, small densities of doped NCs are to be expected through reaction kinetics^33^. For PbS-NCs for example, an excess of Pb during synthesis can potentially lead to a small fraction of *n*-doped PbS NCs, and exposure of PbS NC semiconductors to oxygen to *p*-doping.

**Fig. 2:**
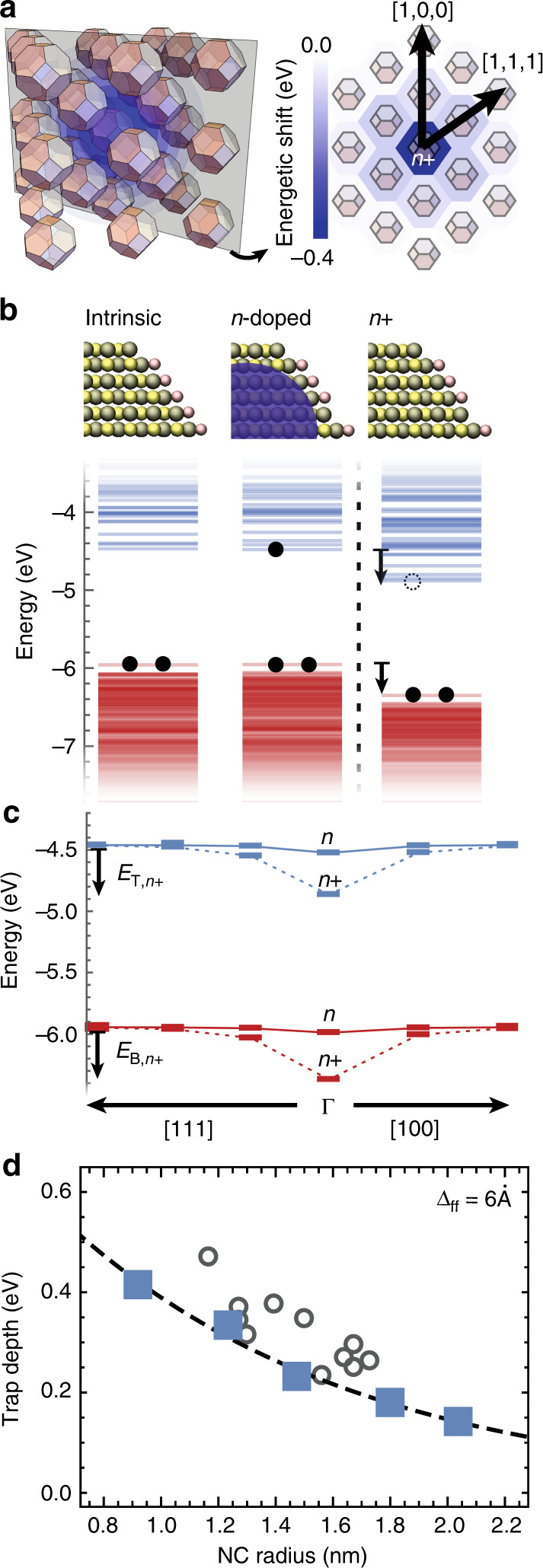
Origin of deep traps in NC semiconductors. **a** A schematic representation of an *n+* or *p*− NC in a solid of intrinsic NCs, where the shift in the energy structure will be screened by neighboring NCs, and a contour plot of the energy shifts of the lowest unoccupied electronic level in NCs (*r* = 0.95 nm with Δ_ff_ = 0.6 Å) for an *n+* NC in an NC solid. **b** Electronic structure of intrinsic, *n*-doped, and oxidized *n*-doped NC (*n+*). **c** Highest occupied electronic levels (red) and lowest unoccupied electronic levels (blue) of an *n*, *n+* NC at the *Γ* point in the BCC lattice and its intrinsic neighbors in the [111] and [100] directions in an NC solid. **d** Trap depth as a function of NC size for NCs in vacuum (circles) and for NC solids (squares). Experimentally measured trap depths on PbS NC solids^23,28^ (gray circles) and the NC charging energies calculated for a sphere of radius *r* in a PbS NC solid (dashed gray line) (see Supplementary Information Note 5) .

We compute the electronic structure of an NC semiconductor containing a single *n-*doped NC surrounded by intrinsic NCs (Fig. 2a). For reference, the electronic structures of isolated intrinsic, *n*-doped, and oxidized *n*-doped NC (with net charge *e+*) are shown in Fig. 2b. In an NC semiconductor, if the *n*-doped NC is not oxidized, its energy levels remain aligned with those of the neighboring intrinsic NCs (Fig. 2c). However, oxidation causes a shift in the energy levels of the *n*-doped NC as well as its neighbors (Fig. 2c, Supplementary Fig. 3).

Within an NC semiconductor, oxidized *n*-doped NCs thus behave as electronic traps for electrons and as barriers for hole transport. Equivalently, reduced *p*-doped NCs present traps for holes and barriers for electrons (Supplementary Fig. 4). Defining the trap depth (*E*_T_(*r,* Δ_ff_)) as the extent of the shift of energy level in an oxidized or reduced doped NC relative to an NC infinitely far from the doped NC (Fig. 2d, Supplementary Fig. 5), we find that our calculated trap depths agree with the experimentally measured trap depths^23,28^ as well as the charging energy of the NCs computed using the measured size-dependent dielectric constant for PbS NC semiconductors^34^. Thus, while trap states have been typically ascribed to mid-gap electronic states on individual NCs, traps will also be presented by the presence of charged, doped NCs in an NC semiconductor.

In this picture, trapping and release of charge carriers from traps is thus simply phonon-assisted charge transfer between the highest occupied or lowest unoccupied states of neighboring NCs with rates given by Eq. (1), where *E*_T_(*r,* Δ_ff_) is included in Δ*E*. Doing so results in release rates on the order of 10^2^ s^−1^ for *r* = 1 nm NCs and up to 10^8^ s^−1^ for *r* = 3 nm, in agreement with the rates characterized previously with thermal-admittance spectroscopy^23^ (see Supplementary Fig. 6).

These results also indicate that the excess carrier on a doped NC must overcome a large energetic barrier (equal to *E*_T_) to become a free carrier in the NC semiconductor. Particularly in small NCs, where *E*_T_ is large, free carrier densities in NC-based semiconductors will be negligible even when large densities of doped NCs are present. For example, for a semiconductor made of *r* = 1.6 nm PbS NCs, assuming 1% of NCs are *n*-doped, the free electron density at room temperature will be ~10^12^ cm^−3^, in stark contrast to the total density of *n*-doped NCs, ~10^16^ cm^−3^. However, the formation of a space-charge region can lead to oxidation (or reduction) of the doped NCs, resulting in high trap densities.

To summarize, our calculations provide several key insights into charge transport in semiconductors assembled from NCs: (1) charge on individual NCs forms polarons, (2) charge transport occurs via phonon-mediated charge transfer, and (3) oxidized or reduced doped NCs become electronic traps states within the NC semiconductor.

### Experimental validation of charge transport and trapping models

**Fig. 3:**
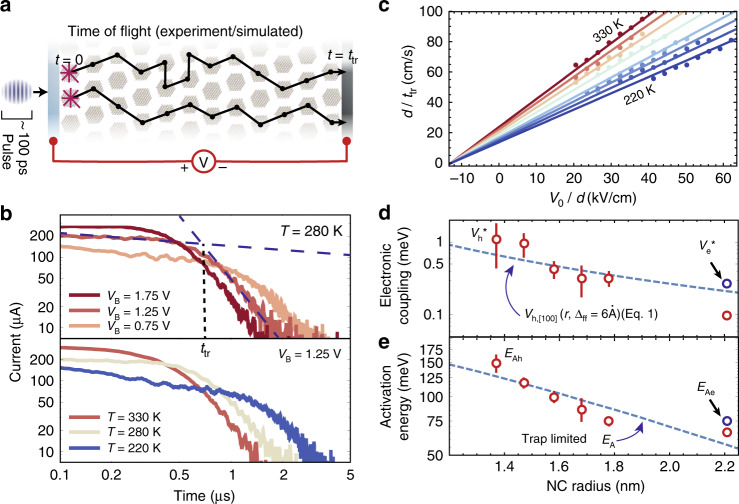
Trap-limited transport in NC semiconductor. **a** Schematic of time-of-flight (TOF) photocurrent transient measurements. **b** Hole transients measured at various biases and temperatures on an NC solid (*r* = 2.21 nm). **c** Plot of the hole velocity, *d*/*t*_tr_, versus applied field, *V*_B_/*d*, at various temperatures. Solid lines indicate the fit to Eq. (4). **d** Electronic coupling and **e** activation energy extracted from TOF measurements as a function of the NC radius *r* compared to computed *V*_h[100]_ and *E*_A_ assuming trap-limited transport (dashed lines); error bars correspond to the standard error in the extracted V^*^_h_ and *E*_Ah_.

We experimentally validate these insights into charge transport in NC-based semiconductors by performing time-of-flight (TOF) photocurrent transient measurements^4^ (see Methods, Supplementary Fig. 7). In a TOF measurement, a laser pulse generates a low density of charge carriers in the NC semiconductor (Fig. 3a), and the displacement current generated by the electrons or holes traversing the film of thickness *d* is measured for a range of biases across the device, *V*_B_, at temperatures *T* ~220–330 K (Fig. 3b). The resulting transients can be fit with two distinct power laws at short and long times (Fig. 3b), with their intersection taken as an effective transit time, *t*_tr_(*V*_B_,*T*). *t*_tr_(*V*_B_,*T*) corresponds to the maximum of the statistical distribution of carrier transport times across the device, and, by fitting *t*_tr_(*V*_B_,*T*) simultaneously for all temperatures *T* and biases *V*_B_ (Fig. 3c), it is possible to extract an effective mobility, *μ*_eff_:

3 $$\frac{d}{{t_{{\mathrm{tr}}}\left( {V,T} \right)}} = \mu _{{\mathrm{eff}}}\left( {r,\Delta _{{\mathrm{ff}}},T} \right)\frac{{\left( {V_{\mathrm{B}} + V_{{\mathrm{B}}0}} \right)}}{d},$$

where *V*_B0_ is the built-in field in the device. The long-time portion of the transient reflects the large dispersion in carrier transit times, and are discussed further in Supplementary Information Note 6.

We first note that, in the limit that the potential drop across neighboring NCs is smaller than the reorganization energy ((*V*_B_ + *V*_B0_)(*2r* + Δ_ff_)*/d* << *λ*), we can expand Eq. (1), and keeping only the term linear in *V*_B_, write:

4 $$\mu _{{\mathrm{eff}}}\left( {r,{{\Delta }}_{{\mathrm{ff}}},T} \right) = N_{\mathrm{p}}\frac{{2{\uppi}}}{\hbar }\frac{{V_{{\mathrm{ct}}}^2}}{{\lambda ^{1/2}}}\frac{{\left( {2r + \Delta _{{\mathrm{ff}}}} \right)^2}}{{2k_{\mathrm{B}}T}}\sqrt {\frac{1}{{4{\uppi}k_{\mathrm{B}}T}}} {\mathrm{e}}^{ - E_{\mathrm{A}}/k_{\mathrm{B}}T},$$

and thereby extract *V*_ct_ and *E*_A_ from experiment (Supplementary Fig. 8). We find good agreement between the experimentally extracted *V*_e_^*^ and *V*_h_^*^ and computed values for electron and hole coupling in the [100] direction (Fig. 3d). The extracted activation energies, which range from 70 to 150 meV (Fig. 3e), are larger than those expected for an NC semiconductor with no energetic disorder (*E*_A_ = *λ*/4 ~ 10 –40 meV) (Fig. 1d). Instead, the activation energies are consistent with the values expected when electronic traps dominate the time scales of carrier transport. The fact that the electronic coupling *V*_ct_ measured in this trap-limited transport regime agrees with our calculations of *V*_ct_ between neighboring NCs indicate that the trap states limiting the effective mobility in NC-based semiconductors are those stemming from oxidized (or reduced) doped NCs.

### Predictive model for charge transport

**Fig. 4:**
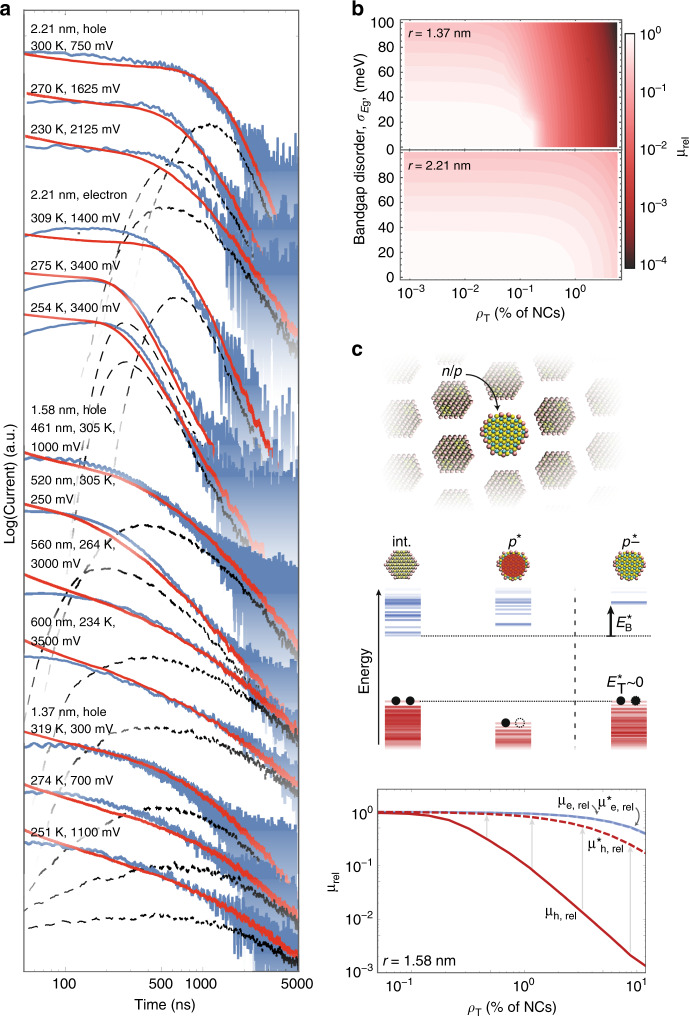
Predictive model for charge transport. **a** Measured TOF transients (blue) and Kinetic Monte Carlo (KMC) simulated transients (red) are shown for various NC sizes, temperatures, biases, and device thicknesses. Distribution of carrier transit times from the simulated transients (dashed black line). **b** Plot of the ratio of the effective mobility as a function of trap density *ρ*_T_ and NC band gap disorder *σ*_*E*g_ to the effective mobility of a trap and disorder-free NC solid for smaller (top) and larger (bottom) NC sizes, calculated for a 400-nm-thick NC solid at 300 K. **c** Doping without the formation of trap states can be achieved by introduction of larger-band gap, *n*- or *p*-doped NCs. Simulations demonstrate that this prevents a decrease in effective mobility at high carrier concentrations.

Confident in our new understanding of charge transport in NC-based semiconductor, we build a Kinetic Monte Carlo (KMC) simulation of polaron transport, which we parameterize with the DFT calculated values for electronic coupling *V*_ct_, reorganization energy *λ*, and electronic trap depth *E*_T_ (see Methods). In this multiscale model, charge transport across an NC-based semiconductor is simulated as sequential charge transfers between neighboring NCs *i* and *j*. The rate of charge transfer is *k*_*ij*_ (which is given by Eq. (1)) with energy offset between neighboring NCs, Δ*E*_*ij*_, is taken as

5 $${\mathrm{\Delta }}E_{ij} = ( {E_{{\mathrm{g}},j} - E_{{\mathrm{g}},i}} )/2 - {\mathbf{E}}_{{z}} \cdot ( {{\mathbf{r}}_j - {\mathbf{r}}_i} ),$$

where *E*_g*,i*_ is the band gap of NC *i*, **E**_*z*_ is the electric field across the NC semiconductor (assumed to be in the *z*-direction), and **r**_*i*_ are the coordinates of NCs. For our simulation, we construct artificial NC semiconductors having the thicknesses and containing the different sized NCs that are investigated experimentally with TOF, and simulate current transients for different biases, carrier types (electrons and holes), and temperatures. Only the density of trap states as a function of NC size, *p*_T_(*r*), is left as a free parameter (Supplementary Information Note 7). The examples shown in Fig. 4a highlight that all simulated transients (red lines) match the measured transients (blue lines), both the effective mobilities defined by *t*_tr_(*V*_*B*_,*T*), as well as the long-time (*t*  > *t*_tr_(*V*_B_,*T*)) dispersion of the transients. We find trap states densities selected to achieve agreement are within the expected range.

## Discussion

This predictive, multiscale model can be used to systematically design next-generation NC-based semiconductors. Here, we consider how to overcome one intrinsic limitation we identified, namely the one-to-one correspondence between the free carrier generation in an NC and the formation of deep traps.

In Fig. 4b, we plot the simulated relative mobility of a PbS NC semiconductor, (*μ*_eff_/*μ*_0_), defined by the time required for ~63% of the carriers to traverse a 400-nm-thick film, as a function of band gap disorder *σ*_*E*g_ and trap density *ρ*_T_, relative to that for a trap- and disorder-free NC semiconductor, *μ*_0_. While band gap disorder has a similar impact on carrier mobility for both small and large NCs, deep traps are more detrimental to transport for small NCs. Their impact can most easily be mitigated by using larger NCs, or by significantly decreasing Δ_ff_ (e.g. through epitaxially connected NC semiconductors^11^) since *E*_*T*_(*r,* Δ_ff_) decreases with increasing *r* and decreasing Δ_ff_).

Our insights enable us to identify a more flexible approach: an NC semiconductor composed of intrinsic NCs can be doped with *p*- or *n*-doped NCs with band gaps larger than that of the intrinsic NCs. With proper selection of band gap, the shifted highest occupied state of oxidized *p*-doped NCs or the lowest unoccupied state of reduced *n*-doped NCs will align with the highest occupied states or lowest unoccupied states of the intrinsic NCs (Fig. 4c). This simultaneously eliminates deep traps and energetic barriers for thermal release of carriers, and leads to multiple orders of magnitude higher mobilities and free carrier densities. Such a strategy can be achieved with a bimodal size distribution of NCs or with equal-sized doped NCs of a different core material (and thus different band gap).

In summary, our insights highlight the need to reframe how we think about charge transport, trapping, and doping in NC semiconductors. As previously discussed, we should expect to find large reorganization energies for any small NCs with X-type ligands, and the formation of trap states upon charging of doped NCs should similarly occur. The approaches employed here can be readily adapted in order to parameterize other NC semiconductors. However, in addition to their size dependence, the reorganization energy and electronic coupling in an NC semiconductor should also depend on the NC material and surface termination, and our finding of small polaron hopping (with *λ* >> *V*_ct_) in PbS systems should not be expected to hold for all NC semiconductors. Indeed, recent characterization of transport in NC semiconductors fabricated with large (~10+ nm) HgTe NCs have provided evidence pointing towards carrier delocalization over subdomains of the NC semiconductor^35^. Finally, we note that the modeling here assumes negligible exciton polarization across individual NCs in the presence of an applied field. For large fields and/or weak confinement, both the reorganization energy and electronic coupling will become field dependent, effects which would need to be accounted for.

Within the polaron hopping regime, NCs semiconductors present highly tunable systems that offer complete control of electronic coupling through tuning of the electronic confinement in the individual NC, the spacing, and the topology of the NC lattice, as well as the activation energies associated with transport through tuning of the NC dispersity, doping, and surfaces. By controlling the phonon densities-of-states and electron–phonon coupling through atomic engineering of the NCs and their surfaces, the rates and temperature dependences of transport can also be systematically tuned. The example of PbS NC-based semiconductors illustrates how it is possible to engineer electronic anisotropies into semiconductors (i.e., transport in [100] will be faster than in [111]), without resorting to anisotropic crystal structures. This enables the creation of semiconductors with isotropic optical properties but with highly anisotropic electronic properties (as in the case of PbS), or with highly anisotropic optical properties and highly isotropic electronic properties. These findings position NC semiconductors not only as highly tunable, solution-processed semiconductors but also as model, tunable systems for studying the fundamental physics of charge transfer processes.

## Methods

### Atomistic model construction for DFT calculations

Atomistic models for the NCs are constructed according to the atomistic model proposed by Zherebetskyy et al.^17^. Bulk rocksalt PbS (with a Pb or S atom centered on the origin) is cut along the eight (111) planes and six (100) planes at plane to origin distances (*r*) defined by the Wulff ratio *R*_W_

6 $$\begin{array}{*{20}{c}} {r_{(1,0,0)} = AR_{\mathrm{W}},} & {r_{(1,1,1)} = AR_{\mathrm{W}}^{ - 1}.} \end{array}$$

A *R*_W_ = 0.82 is used^17^. The scalar *A* is adjusted such that the resulting NC is S-terminated on the (111) facets. These (111)-surface terminating S atoms are then replaced with the desired ligand (for all calculations here, iodide anions). To obtain an intrinsic semiconductor NC, overall charge balance must be maintained according to Eq. (4) in the main text. For all NC sizes investigated here, 1–2 ligands or lead–ligand pairs are removed from the as cut atomistic model in order to satisfy charge balance. For the doped NCs, a single ligand or lead–ligand pair is additionally removed. All removed atoms are taken from the corners of the NCs corresponding to the intersection of the [111] and [100] facets.

### Electronic structure and electron transfer parameterization calculations

All electronic structure calculations are performed within the CP2K program suite utilizing the quickstep module^36^. Calculations are carried out using a dual basis of localized Gaussians and plane waves^37^, with a 300 Ry plane-wave cutoff. Double-zeta-valence polarization^38^, Goedecker–Teter–Hutter pseudopotentials for core electrons, and the Perdew–Burke–Ernzerhof (PBE) exchange correlation functional are used for all calculations, as in previous calculations for PbS NCs^16,23^. Convergence to 10^−8^ in self-consistent field calculations is enforced for all calculations unless otherwise specified.

Non-periodic boundary conditions in atomic coordinates and electric potential are used (with the exception of the superlattice calculations which uses periodic boundary conditions for both), through the use of a wavelet Poisson solver^39^. Geometry optimization is performed with the Quickstep module utilizing a Broyden–Fletcher–Goldfarb–Shannon optimizer. All atoms in all systems are relaxed using maximum force of 24 meV Å^−1^ as convergence criteria.

### Reorganization energy calculations

Reorganization energies are calculated using a half- cell approach^40^. We first fully geometrically relax the atomic coordinates, *Q*_*x*_, and compute the total energy of the neutral NC, *E*_n_(*Q*_n_), the NC with an additional electron, *E*_e_(*Q*_e_), and an NC with a hole, *E*_h_(*Q*_h_). We then perform energy calculations, without any geometry optimization, for *E*_n_(*Q*_e_), *E*_n_(*Q*_h_), *E*_h_(*Q*_n_), *E*_e_(*Q*_n_). Then,

7 $$\begin{array}{l}\lambda _{\mathrm{h}} = E_{{\mathrm{Tot}}}\left( {{\Psi}_{\mathrm{P}},Q_{\mathrm{R}}} \right) - E_{{\mathrm{Tot}}}\left( {{\Psi}_{\mathrm{P}},Q_{\mathrm{P}}} \right) = \left( {E_{\mathrm{n}}\left( {Q_{\mathrm{h}}} \right) + E_{\mathrm{h}}\left( {Q_{\mathrm{n}}} \right)} \right) - \left( {E_{\mathrm{n}}\left( {Q_{\mathrm{n}}} \right) + E_{\mathrm{h}}\left( {Q_{\mathrm{h}}} \right)} \right)\\ \lambda _{\mathrm{e}} = E_{{\mathrm{Tot}}}\left( {{\Psi}_{\mathrm{P}},Q_{\mathrm{R}}} \right) - E_{{\mathrm{Tot}}}\left( {{\Psi}_{\mathrm{P}},Q_{\mathrm{P}}} \right) = \left( {E_{\mathrm{n}}\left( {Q_{\mathrm{e}}} \right) + E_{\mathrm{e}}\left( {Q_{\mathrm{n}}} \right)} \right) - \left( {E_{\mathrm{n}}\left( {Q_{\mathrm{n}}} \right) + E_{\mathrm{e}}\left( {Q_{\mathrm{e}}} \right)} \right).\end{array}$$

### Electronic coupling

These calculations are performed on a system of two NCs, oriented according to the two configurations in Fig. 2 of the main text, for all NC sizes, *r*, and a range of facet-to-facet separations, Δ_ff_. *V*(*r*, Δ_ff_) for the CBM and VBM is then taken as half the splitting of the resulting antisymmetric and symmetric states in the combined system^41^.

### Superlattice electronic structure calculations

The electronic structure of the NC superlattices were calculated utilizing the Kim–Gordon method (KG), which partitions a weakly interacting system into subunits. Namely, it forces the overall Hamiltonian of the superlattice (the weakly interacting system) to be block diagonal, where each block corresponds to a strongly interacting subunit (each individual NC). The KG approximation should be very much suitable for the NC superlattices, given our finding of weak electronic coupling between the NCs, and localization of charge carriers on individual NCs as a result of the large reorganization energies associated to polaron formation. A detailed description of the KG method can be found elsewhere^42^. For the calculations here, a linear-scaling approach to self-consistent field was employed, using a full embedding potential for the nonadditive kinetic energy correction to the PBE functional.

Calculations are performed on 5 × 5 × 5 structures (corresponding to ~200,000 atoms for the largest NC), as calculations as a function of superlattice size indicate a convergence of the trap depth with this size (see Supplementary Fig. 2). In Supplementary Fig. 2 we additionally plot the trap depth for the 0.9 nm NC as a function of Δ_ff_, which, as expected, indicates an increase in trap depth with an increase in NC–NC separation resulting from weaker screening.

### Kinetic Monte Carlo simulations

The Kinetic Monte Carlo simulations are performed within the limit of low charge carrier concentration, which assumes negligible interaction between the charge carriers. Charge transport is then simulated as sequential charge transfers (CT) between NCs. The charge transfer rate between two NCs *i* and *j*, *k*_*ij*_, is given by Eq. (1) in the main text,

8 $$k_{ij} = N_{\mathrm{P}}\frac{{2\pi }}{\hbar }V_{ij}^2\sqrt {\frac{1}{{4\pi \lambda k_{\mathrm{B}}T}}} {\mathrm{exp}}\left[ { - (\Delta E_{ij} + \lambda )^2/4\lambda k_{\mathrm{B}}T} \right],$$

where Δ*E*_*ij*_ is the energy of the products minus the energy of the reactants, and *N*_P_ is the number of degenerate product states. We take Δ*E*_*ij*_ as

9 $$\Delta E_{ij} = \frac{{E_{\mathrm{g,j}} - E_{\mathrm{g,i}}}}{2} - E_z({\mathbf{r}}_j - {\mathbf{r}}_i) \cdot {\mathbf{z}},$$

where *E*_g*,i*_ is the band gap of NC *i*, *E*_*z*_ is the electric field across the NC solid (assumed to be in the *z*-direction), and *r*_*i*_ is the coordinate of NC *i*.

For PbS NCs, intervalley coupling which stems from the [100] facets of the NC, break the fourfold degeneracy of the valence band maximum (VBM) and conduction band minimum (CBM) of bulk-PbS in the NCs. This results in NC conduction band minima and valence band maxima of which are either singly or triply degenerate. This splitting is discussed in detail elsewhere^16^, but we find the ordering to vary between differently sized NCs, i.e. for some sizes the VBM/CBM are singly/triply degenerate, whereas for other sizes, they are found to be triply/singly degenerate. We therefore use an average of *N*_P_ =  2 for both electron and hole transfer.

If a charge carrier is assumed to be on NC *i*, the NC to which it hops, and the time required for that hop to occur are determined in the following way^43^: First, the CT rates, the set {*k*_*j*_}, are computed for a hop from NC *i* to all of its [100] and [111] nearest neighbors, the set {*j*}, according to Eq. (M3). Next, the hopping time to all nearest neighbors, the set {*t*_*j*_}, are calculated via inverse transform sampling,

10 $$t_j = - \frac{{\ln \left[ {U_j} \right]}}{{k_j}},$$

where *U*_*j*_ is a random number pulled (one for each NN {*j*}) from a uniform distribution between 0 and 1. The smallest hopping time from the set {*t*_*j*_} is then taken as the hopping time, and the hop occurs to the NC, *j*, which corresponds to this smallest hopping time.

### Superlattice construction and TOF simulations

Simulations are performed on a BCC superlattice of NCs with unit cell dimensions 32 × 32 × 1000. The band gaps of each NC in the superlattice are pulled from a normal distribution with a mean of 0, and band gap inhomogeneity is characterized by a standard deviation *σ*_*E*g_. Deep traps are added to the superlattice structure at random, at a density *ρ*_T_, according to the trap depth given by Eq. (5) in the main text and Eq. (M4) in the Supplementary Materials. The levels of each deep traps nearest neighbors are additionally shifted, according to the parameterization presented in Supplementary Materials. We assume that the electrostatic shifts are additive, i.e. two neighboring traps will additionally shift each other’s levels further, according to the parameterization in Supplementary Materials.

A single charge carrier is initialized at time *t* = 0 on a randomly selected NC at *z* = 0. The KMC then proceeds by sequentially hopping the carrier from NC to NC according to the procedure outlined above. The simulation is then terminated once the charge carrier reaches a *z* value corresponding to the device thickness. The result of the simulation is a set of charge carrier arrival times at a given NC coordinate, {*t*_*j*_, *r*_*j*_}, from which we can compute the TOF measured displacement current,

11 $$I(t_{j - 1} < t \le t_j) = e\frac{{({\mathbf{r}}_j - {\mathbf{r}}_{j - 1}) \cdot {\mathbf{z}}}}{{(t_j - t_{j - 1})d}},$$

where *d* is the device thickness. This procedure is carried out stochastically for 10^5^ charge carriers and the results averaged for the overall TOF transient, generating a uniquely disordered NC superlattice for each charge carrier simulated.

### PbS synthesis

Colloidal oleic acid capped PbS NCs are synthesized using the hot injection method^44,45^. ﻿Briefly, 80 mmol of PbO is combined with 70 mL of oleic acid in 730 mL of 1-octadecene. Forty millimoles of bis(trimethylsilyl)sulfide in 400 mL of dried 1-octadecene is then rapidly injected into the PbO solution at 150 °C under vacuum. A three-stage cooling procedure is then followed with (1) natural cooling down to 100 °C (approximately 4 min duration) (2) the solution is held at 100 °C for 5 min (3) the reaction is then terminated with a large ice-water bath. The size of NCs are tuned by adjusting the concentration of oleic acid in the PbO/1-octadecene mixture. Post synthesis NCs are washed three times in mixtures of ethanol and methanol, and finally suspended in hexane at a concentration of 40 mg/mL. We determine the size of the NCs from their absorption spectrum using a well-established parametric model^4^.

### PbS NC-layer fabrication

PbS NC layers were fabricated on substrates described below through sequential dip coating in (i) PbS NC solution diluted to 5 mg/ml in hexane, (ii) crosslinking solution of 6 mM ethanedithiol (EDT) in anhydrous acetonitrile, and (iii) rinsing solution of anhydrous acetonitrile. Dip coating was carried out in air. The thickness of the PbS-NC layers were adjusted by the number of dip-coating cycles, and thicknesses were measured from SEM cross-sections of the devices after characterization, using ~100 measurements of the thickness over a cross-section spanning the entire device.

### Device fabrication

For the standard heterojunction devices, a TiO_2_ nanoparticle paste (DSL 90-T, Dyesol) diluted to 125 mg/mL in acetone was spun on fluorinated tin oxide/glass substrates (Solaronix) at 70 × *g* for 60 s. Samples were annealed on a hotplate at 500 °C for 60 min, then immersed in a 60 mM titanium-tetrachloride/deionized water solution at 70 °C for 30 min, and then placed on a hotplate at 500 °C for 60 min after thorough rinsing with deionized water. Top contacts of MoOx/Au/Ag (20, 100, and 500 nm) electrodes were deposited by thermal evaporation. For the inverted heterojunction devices, NiO was deposited by RF magnetron sputtering using a 99.95% purity NiO target onto indium tin oxide (ITO) glass substrates (Thin Film technologies) using a 10% partial pressure of oxygen. Top contacts of LiF/Al/Ag (10, 100, and 500 nm) electrodes were deposited by thermal evaporation.

### Time of flight measurements

Samples are mounted into a cryostat (Janis ST-500) and remain in vacuum during the measurements. The cryostat is mounted on a Nikon Eclipse Ti-U optical microscope. A 405 nm, 100 ps excitation pulse is provided by a Hamamatsu picosecond pulsed laser (PLP-10). We note that the excitation energy is larger than the band gap of the NCs; however, this should not impact the transient dynamics as PbS NCs have hot-carrier cooling times on the order of 100 s of fs. Voltage biases were applied using an Agilent 33522A arbitrary waveform generator and the current was measured on a Rohde&Schwarz RTM1054 oscilloscope through the 50 Ω input. Measurements were averaged over 1024 cycles at a frequency of 10 kHz.

## Supplementary information

Supplementary Information

## Data Availability

The datasets generated during and/or analyzed during the current study are available from the corresponding author on reasonable request.
