# Supplementary material for: Charge transport in semiconductors assembled from nanocrystal quantum dots
Source: Nat Commun. 2020 Jun 5;11:2852. doi: 10.1038/s41467-020-16560-7 (PMC7275058; doi:10.1038/s41467-020-16560-7)
Supplement: Supplementary file 1 — Supplementary Information [file 41467_2020_16560_MOESM1_ESM.pdf]

Supplementary Information

Charge Transport in Semiconductors Assembled from Nanocrystal Quantum Dots

Yazdani *et al.*

Supplementary Note 1 - Polaron Formation and Associated Reorganization Energy

Calculations were carried out and reorganization energies were calculated as described in the material and methods. Here we provide further analysis of the structural deformations associated with charging of the NC with an electron or hole. We compute the change in Pb-ligand and Pb-S bond lengths of the NCs upon charging with an electron or hole,

$$\Delta_{\text{Pb-X}} = \frac{1}{N_{ij}} \sum_{\{i,j\}} \left(\left| \mathbf{q}_{\text{n},\text{Pb}_i} - \mathbf{q}_{\text{n},X_j} \right| - \left| \mathbf{q}_{c,\text{Pb}_i} - \mathbf{q}_{c,X_j} \right| \right), \quad (1)$$

where X = {Lig, S}, the sum over $\{i,j\}$ runs over all nearest neighbor pairs of Pb-X, and $\mathbf{q}_{c,\text{Pb}i}$ and $\mathbf{q}_{c,Xi}$ are the vector coordinates of the Pb and Lig/S atoms in the charge state c = {n, e,h} from the nuclear coordinates $\{Q_\text{n}, Q_\text{e}, Q_\text{h}\}$. The results for the PbS-I NC as a function of r are shown **Supplementary Figure 1**. We find a nearly symmetric extension/contraction of the average PbS-I bond lengths upon charging with an electron/hole, with far smaller changes in the Pb-S bond lengths, so small that they are below the accuracy of the calculations.

The high electronegativity of the ligands ensures that they will be strongly negatively charged relative to the Pb and S atoms, and that electrostatic interaction of the charge carrier with the ligands is the main mechanism driving the polaron formation. This suggests that the choice of ligand, and its effective electronegativity. To investigate this further, we repeated the calculations on a Cl terminated NC, with its results plot in **Supplementary Figure 1**. Although the Cl electronegativity (3.16) is significantly larger than I (2.66) the overall shift in Pb-Cl bond lengths are similar to those for the PbS-I NC, with a slight average increase in the corresponding reorganization energy. We can thus expect only slight variations in the polaron mediated reorganization energies for commonly employed ligands, e.g. thiols (effective electronegativity of 2.45)[1]. We note that, while it would be preferential to directly compare to calculations on other classes of ligands (e.g. thiol, carboxylate), the large number of nuclear degrees of freedom of complex organic ligands restricts one's ability to identify with sufficient accuracy the nuclear ground state of the NC, rotations of the ligands being particularly problematic. It is therefore unfortunately impractical to directly compute the reorganization energy with the method employed here. We however stress that due to the Pb-rich character of PbS-NCs and the large effective electronegativities of all employed ligands, that the electrostatic mechanism driving polaron formation will persist, and that our I-Cl comparison suggests that the scale of the associated reorganization energies should remain similar.

Supplementary Note 2 - Outer-Shell Reorganization Energy

Throughout the work in the main text, we ignore any contribution from outer-shell reorganization. Here we motivate that approximation, as well as comment on the common assumption in literature that the charging energy of the NC contributes to the energetic barrier associated with charge transport.

The reorganization energy associated with charge transfer, λ, is often split into two components, the outer-shell-, λ_o, and inner-shell-, λ_i, reorganization energies [2],

$$\lambda = \lambda_\text{o} + \lambda_\text{i}. \quad (2)$$

λ_i is associated with the reorganization of internal nuclear coordinates of the reactants/products themselves upon charge transfer, while λ_o is associated with the reorganization of the nuclear coordinates of the surrounding medium. Unfortunately, direct calculation of the total reorganization energy, λ, for charge transfer in a NC-solid is currently out of reach computationally, requiring full geometric relaxation of systems with ~10^5+ atoms. In the main text, we make the approximation that

$$\lambda \simeq \lambda_i^{'}, \tag{3}$$

where here we use the notation $\lambda_i^{'}$ to indicate the inner-shell reorganization energy computed assuming that charge transfer occurs between the two NCs in isolation, i.e. no surrounding medium.

Electrostatic interaction of the charge carriers on the individual NCs with the negatively charged ligands leads to an extension/contraction of the lead ligand bonds. We can therefore expect an analogous effect to occur on the NCs neighboring the reactants and products within the NC solid, namely, that the presence of a charge carrier on a NC will lead to slight shifts of the Pb-Ligands on its neighboring NCs. However, these interaction have a significantly longer range relative to the interactions with the ligands on the charged NC, and these long ranged interactions are furthermore already screened by the polaron formation on the charged-NC, we can therefore expect the outer-shell reorganization energy to be significantly smaller than the inner-shell, $\lambda_o < \lambda_i$.

In the case of organic ligands, reorientational motion of any dipole moments on the ligands can further contribute to the outer-shell reorganization energy. Indeed, capacitive measurements on NC-solid thin films typically show a monotonously increasing capacitive response at low frequencies (< 10^4 Hz),[3,4] followed by a flat response at higher frequencies. Given that the charge transfer timescales (ps – μs including release from deep traps) are significantly faster than these slow processes, we can safely ignore their contribution to the reorganization energy.

Finally, we comment on the common assumption that the activation energy associated with charge transport corresponds to the charging energy of the NC, i.e. the energy required to oxidize/reduce a NC, striping a charge carrier from the NC and bringing it infinitely far away. There is a fundamental problem with this picture, in that it assumes an intermediated state during the process of a carrier hop in which the overall NC-solid is charged. However, during the charge-transfer process, the charge carrier is never temporarily 'pulled out into vacuum', but rather undergoes a phonon mediated tunneling between the two NCs. We nevertheless acknowledge the appeal of assigning the activation energy measured experimentally to the charging energy, indeed, as we have shown, charge transport in many NC-solid systems will be limited by deep-traps, whose trap depth is precisely the charging energy!

Supplementary Note 3 - Parameterization of Electronic Coupling

Calculations of the electronic coupling between pairs of NCs as a function of NC radii, r, and inter-NC facet-to-facet distances, Δ_{ff}, were performed as described in the material and methods described above. Due to the exponential form of the gaussian basis functions used in the DFT at large distances, calculations were performed for facet to facet distances from ~3.6-5.4 Å, and then extrapolated to 6 Å. The points which are plot in Figure 2 of the main text correspond to the extrapolated coupling at 6 Å, determined by fitting the couplings separately for each NC size and

orientation with an exponential function. For the final fit presented in **Supplementary Table 1**, all points were simultaneously fit.

Supplementary Note 4 – Transition Rates

A general expression for the transition rate is given by Lin *et al.*,[5]

$$k = \frac{1}{\hbar^2}\left|V_{\mathrm{ct}}\right|^2 \int_{-\infty}^{\infty} dt \exp\left[it\left(\Delta E/\hbar\right) - \sum_j S_{\omega_j}\left[\left(2n_j+1\right) - n_j e^{-it\omega_j} - \left(n_j+1\right)e^{it\omega_j}\right]\right], \tag{4}$$

which is written in terms of the Huang-Rhys parameter S_{ω_j}, which is related to the reorganization energy through the relation

$$\lambda = \sum_j S_{\omega_j}\hbar\omega_j, \tag{5}$$

where the sum is over all phonon modes which couple to the transition. **Supplementary Equation 4** reduces to eq. 1 in the main text provided that $k_B T$ is much higher than the energies of all phonons coupling to the transition, i.e. $k_B T \gg \hbar\omega_j$ for all j. A simplified form of **Supplementary Equation 4** can be derived, with the approximation that a single phonon mode couples to the transition,

$$\lambda = \sum_j S_{\omega_j}\hbar\omega_j \equiv S_{\tilde{\omega}}\hbar\tilde{\omega}, \tag{6}$$

in which case, for $k_B T \ll \hbar\tilde{\omega}$,

$$k = \begin{cases} \dfrac{2\pi}{\hbar^2\tilde{\omega}}\left|V_{\mathrm{ct}}\right|^2 e^{-S_{\tilde{\omega}}} \dfrac{S_{\tilde{\omega}}^{\left|\Delta E\right|/\hbar\tilde{\omega}}}{\left(\left|\Delta E\right|/\hbar\tilde{\omega}\right)!}, & \Delta E < 0 \\ 0, & \Delta E > 0 \end{cases} \tag{7}$$

In the case of isoenergetic NCs and an applied electric field, $\Delta E = \vec{E}\cdot\left(\vec{r_j} - \vec{r_i}\right)$, where $\vec{r_j}$ is the position of NC j. The transition rate is therefore temperature independent, and depends non-trivially on the applied field.

Supplementary Note 5 - Further Information on Deep-Trap Calculations

Calculations for the trap-depth as a function of r were performed as described in the methods section. The computed trap depths are plot in **Supplementary Figure 3**, and we find the trap depths over the range of NC radii investigated here can be fit excellently with the expression

$$E_{\mathrm{T}}(r) = E_{\mathrm{T0}}e^{-\beta_{\mathrm{T}}r}, \tag{8}$$

with $\beta_T = 1.0 \pm 0.1$ nm, $E_{T0} = 1000 \pm 100$ meV. Similarly, the electrostatic shift of the levels of the traps nearest neighbors can also be fit with this expression (see **Supplementary Figure 3**). Nearest neighbors in 111 direction have $E_{T0}([111]) = 222 \pm 4$ meV, and in the 100 direction $E_{T0}([100]) = 151 \pm 2$ meV.

The charging energy we plot in Fig. 3e in the main text, E_C. is given by [6]

$$E_C(r, \Delta_{ff}) = \frac{e^2}{2r \cdot \epsilon_R(r, \Delta_{ff})}, \tag{9}$$

where ε_R is the dialectric permittivity of the NC-Solid. We take an empirical fit to the measured ε_R of Grinolds et al. [4], with

$$\begin{aligned} \epsilon_R(r, \Delta_{ff}) &= 1 + 60.45 v_f(r, \Delta_{ff}), \\ v_f(r, \Delta_{ff}) &\equiv p\left(\frac{r^3}{(r + \Delta_{ff}/2)^3}\right), \end{aligned} \tag{10}$$

where v_f is the volume fill fraction of a NC-solid. and p is the packing density ($p = 0.68$ for BCC).

Supplementary Note 6 - Additional TOF Analysis

Impact of spatial and energetic disorder We first consider the impact of energetic disorder of the NC band-gaps on the expected electronic coupling and activation energies extracted from the TOF measurements.

In reality, energetic disorder will be present, in the form of disorder of the NC bandgaps, as a result of inhomogeneous sizes of the individual NCs. We can estimate an upper limit of the energetic disorder from the photoluminescence spectrum of the NCs (see **Supplementary Figure 7**).[1] The band-gaps are normally distributed with standard deviation σ_g. The mean activation energy, $\langle E_A \rangle$, assuming a normal distribution of band-gaps is given by

$$\langle E_A(r) \rangle_x = \frac{\lambda(\mathrm{r})}{4} + \frac{\sigma_g^2}{2\lambda(r)\chi_x^{\ 2}}, \tag{11}$$

where $x = e/h$, and χ_x is a parameter describing the alignment of the HOMOs/LUMOs of NCs with different band-gaps, $\chi_x > 0$, $\chi_e^{-1} + \chi_h^{-1} = 1$. Assuming $\chi_e = \chi_h = 2$, the maximum expected mean activation energies span a range $\langle E_A(2.21\text{nm}) \rangle_h = 21$ meV $\rightarrow$ $\langle E_A(1.37\text{nm}) \rangle_h = 28$ meV, significantly smaller than the extracted E_{Ah}.

We next consider the expected activation energies one would observe with the assumption that release from deep traps is limiting transport. In this case, the observed activation energy would correspond to the mean activation energy required to escape the deep trap. Each NC has 8 nearest neighbors in the [111] directions, and 6 nearest neighbors in the [100] directions, and we approximate that the activation energy required for release will correspond to the lowest activation energy between the deep trap and its neighboring NCs. We stochastically compute the mean activation energy, performed numerically via

$$\langle E_A \rangle = \langle \mathrm{Min}[\{E_{A1}, E_{A2}, \dots E_{A14}\}] \rangle,$$
$$E_{An} = \left(\frac{E_{gT} - E_{gn}}{2} + E_T(r) + \lambda(r) \right)^2 / 4\lambda(r), \tag{12}$$

where E_{gT} is the bandgap of the deep trap NC, and the E_{gn} are the bandgap of its neighbors, all taken from a normal distribution with standard deviation σ_g, $E_T(r)$ is the trap depth. We compute this, averaging over 10^6 stochastic realizations, and the results are plot in **Fig. 3f** of the main text. The expected E_A calculated show excellent agreement with those measured.

Dispersive Charge Carrier Transport and Undefined Mobilities Scaling the transients to their extracted transit times, $t \to t/t_{tr}$ and $I \to I \cdot t_{tr}$, leads to a collapse onto a single curve, which is independent of the electric field and device thickness, d. In **Supplementary Figure 9**, we plot an example of such a collapse for devices fabricated with r = 1.58 nm NCs, for fields 10-100 keVcm^{-1}, devices thicknesses d = 460-600nm, and temperatures T = 235 – 305 K. The long time portions of the transients, $t/t_{tr} > 1$, are fit with a power law with exponent $\alpha(r,T)$,

$$I(t) \propto t^{-\alpha(rT)}. \tag{13}$$

The extracted $\alpha(r,T)$ for several NC sizes are plot in **Supplementary Figure 9**. For smaller NCs, $\alpha(r,T)$ is less than 2 for the entire temperature range, indicated by the red-shaded region in the plot. This indicates highly dispersive carrier transport, with undefined mean carrier transit times. The carrier mobility for these sizes is therefore undefined within this temperature range, one of the consequences being a series resistance which will increase super-linearly with material thickness.

Supplementary Note 7 - Fitting Kinetic Monte Carlo Simulations to Experimentally Measured TOF Transients

Kinetic Monte Carlo simulations are carried out as describe in the materials and methods. As input to the simulations, V_{100}, V_{111}, *and* λ, are calculated from Eqs. 1 and 2 in the main text. V_{B0} is extracted from the experimentally fitted mobilities, eq. 6 in the main text, giving a field $E_z = (V_B + V_{B0})/d$. The bandgaps of the NCs are assumed to be normally distributed with standard deviation σ_g, which we fix to its upper limit represented by the standard deviation of the photoluminescence of the NCs (Fig. SI-3). Deep traps (n+/ p- NCs for electron/holes respectively) are added to the NC-solid by shifting the bands of a random fraction, ρ_T, of the NCs according to the trap depth, E_T, as well as the shift of their neighbors in the 111 and 100 directions, E_{T100}, E_{T111}, calculated from **Supplementary Equation 8**. We ignore energetic barriers (p-/n+ NCs for electron/holes respectively) in the calculations, as the high dimensionality of the NC-solid mitigates their impact on the carrier dynamics. For all samples, we then fit the KMC transients to the experimentally measured TOF transients by seeking a minimum least squared residual between the simulated and measured transients over a broad range of temperatures, biases, and thicknesses. This fitting is accomplished by varying only the density of deep traps for each NC size and carrier type. The fitted density of traps between 0.1-10% are in agreement with measured values.[7] The input parameters for the samples shown in the main text are given in **Supplementary Table 2**.

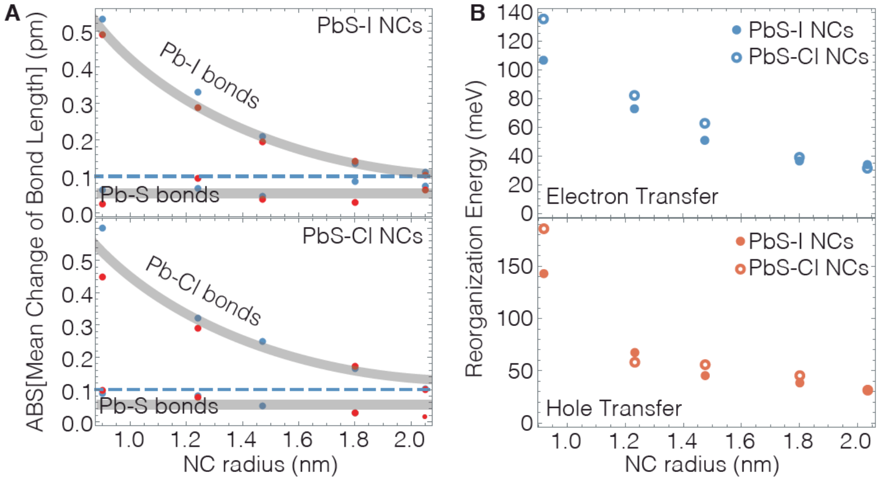

Supplementary Figure 1 – Polaron Formation and Reorganization Energies (A) Plot of the change in Pb-Lig and Pb-S ligands upon charging the NC with an electron (blue) and hole (red) as a function of *r* for PbS-I and PbS-Cl NCs. (B) Associated reorganization energies for electron and hole transfer for both the PbS-I and PbS-Cl NCs.

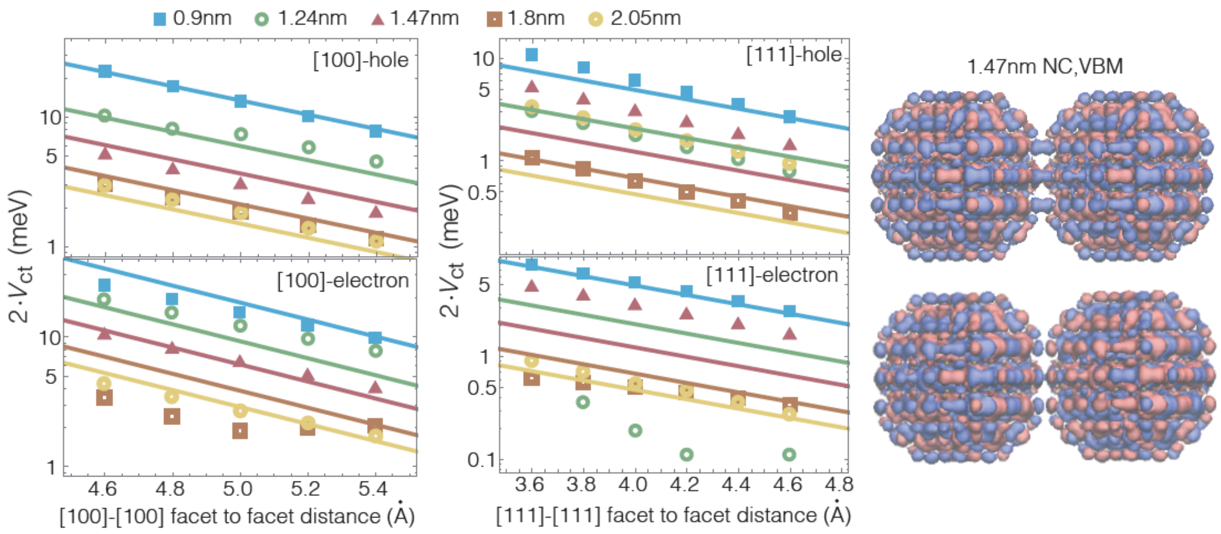

Supplementary Figure 2. **Electronic Coupling** The computed electronic coupling for electrons and holes in both the [100] and [111] NC-NC configurations are shown, along with the fit of **Supplementary Equation 2** of the main text. Here, the x-axis corresponds to the real facet to facet distance of the calculations, which for the [111] configuration is equal to Δff, but not for the [100] To the right, an example of the symmetric/antisymmetric coupled state is shown for the VBM of the 1.47 nm NC

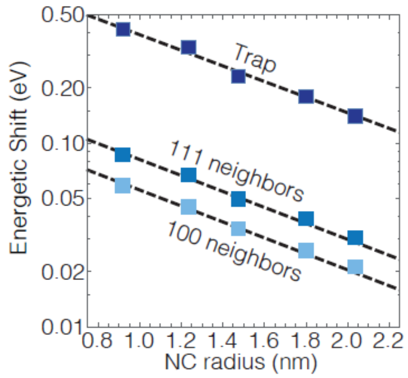

Supplementary Figure 3. Computed trap depth as a function of r with Δ_{ff} = 6 Å, along with the magnitude of the electrostatic shift of the levels of the traps nearest neighbors in the [100] and [111] directions.

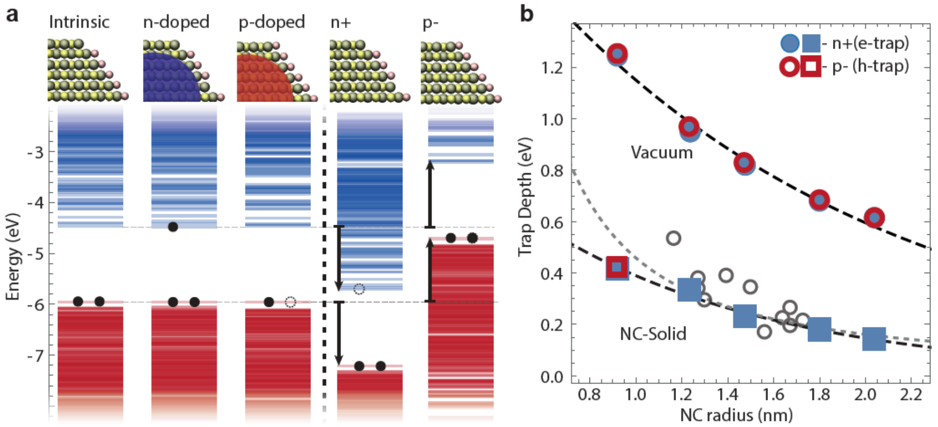

Supplementary Figure 4 Oxidized/Reduced n- and p-doped NCs present Deep Traps for Charge Transport (**A**) Electronic structure of intrinsic, n-doped, and p-doped NCs (r = 0.95 nm) in vacuum. Upon oxidation of an n-doped NC (n+) or reduction of a p-doped NC (p-), the electronic structure shifts. (**B**) Trap depth as a function of NC size for NCs in vacuum (circles) and for NC-solids (squares), experimentally measured trap depths on PbS NC-solids[3,7] (gray circles) and the NC charging energies calculated for a sphere of radius r in a PbS NC-solid (dashed gray line).

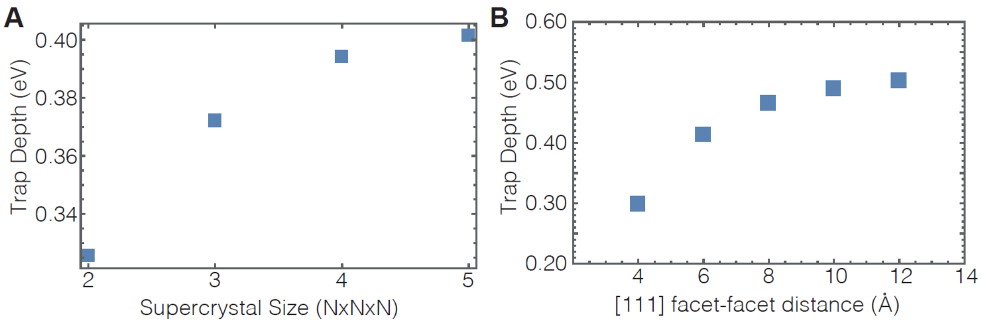

Supplementary Figure 5. **Trap Depths** (A) Trap depth of the smallest NC (~0.9nm) with Δ_{ff} = 6 Å computed for different superlattice sizes, showing a convergence of the trap depth with a 5x5x5 superlattice. (B) Computed trap depths for the smallest NC as a function of Δ_{ff}.

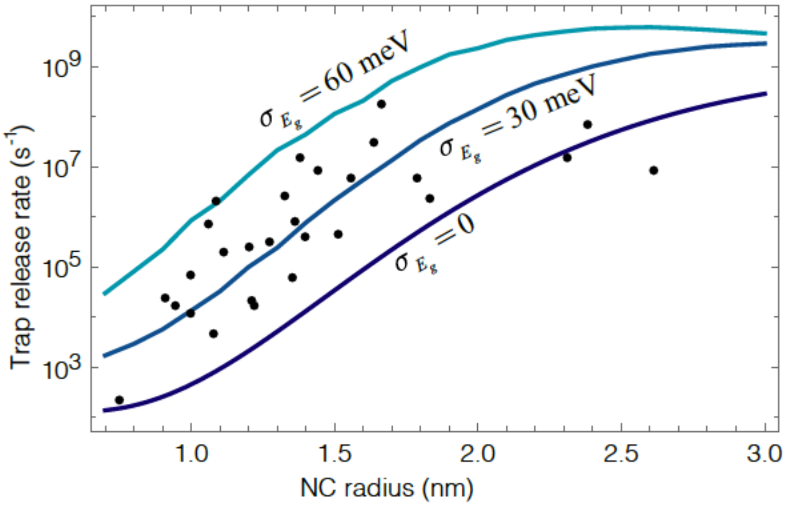

Supplementary Figure 6. **Release Rates from Deep Traps** Previously measured (Ref. 3) release rates from deep traps, along with calculated rates (Equation 1 of the main text) for NC-semiconductors with varying band-gap disorders.

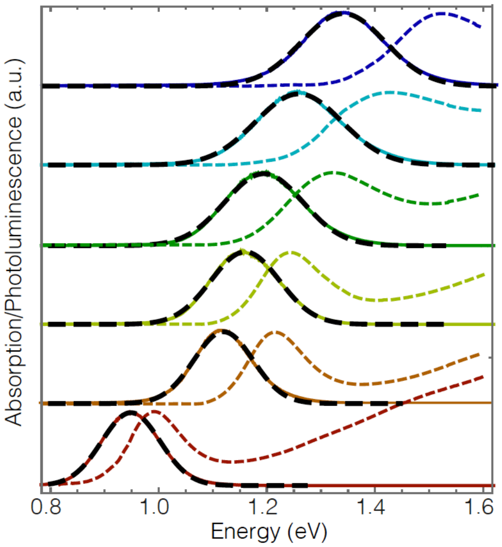

Supplementary Figure 7 – PbS Samples Absorption (dashed colored lines) and luminescence (solid lines) spectra of PbS NC solutions used for TOF characterization. Gaussian fits to the luminescence spectra are indicated by the dashed black lines.

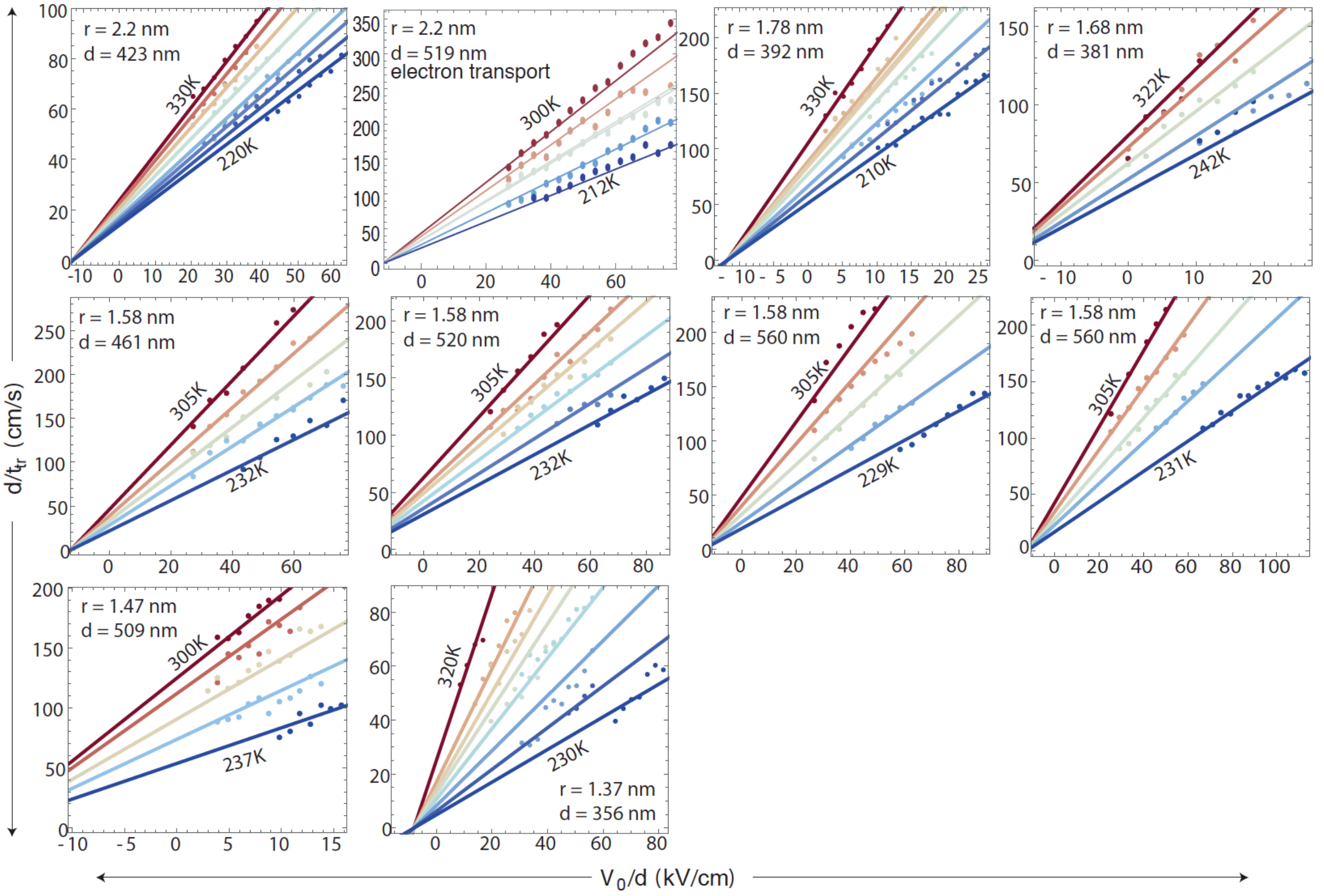

Supplementary Figure 8. Extracted transit times, t_{tr}, as a function of applied field and temperature for all samples investigated in this work, along with the corresponding fits for Equation 4 of the main text.

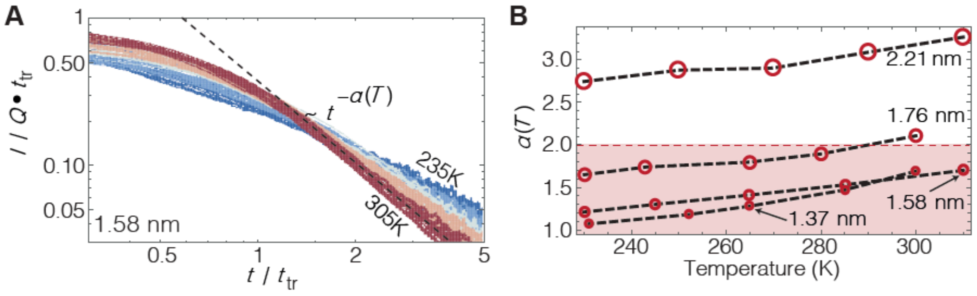

Supplementary Figure 9. (A) Plot of the scaled TOF transients, $t \rightarrow t/t_{tr}$ and $I \rightarrow I \cdot t_{tr}$, for devices fabricated with r = 1.58 nm NCs, for fields 10-100 keVcm^{-1}, devices thicknesses d = 460-600nm, and temperatures T = 235 – 305 K. (B) The extracted $\alpha(r,T)$ as a function of temperature for several NC sizes. The red region indicates $\alpha(r,T) < 2$, i.e. the region of $\alpha(r,T)$ for which the mean transit time is undefined.

Supplementary Table 1. Functional fits to transport parameters.

Quantity	Fit Function	Fit Parameters
Reorganization Energy, λ	$\lambda(r) = \lambda_0 r^{-\gamma}$	$\lambda_0 = 106 \pm 5$ meV·nm$^{\gamma}$<br>$\gamma = 1.9 \pm 0.2$
Electronic Coupling, V_{ct}	$V_x(r, \Delta_{\text{ff}}) = V_{0x} e^{-\beta \Delta_{\text{ff}}} r^{-\eta}$	$\beta = 1.2 \pm 0.1$ Å^{-1}<br>$\eta = 2.4 \pm 0.5$<br>$V_{0,e[111]} = V_{0,h[111]} = 300 \pm 220$ meV·nm$^{\eta}$<br>$V_{0,e[100]} = 3100 \pm 200$ meV·nm$^{\eta}$<br>$V_{0,h[100]} = 1900 \pm 100$ meV·nm$^{\eta}$
Trap Depth, E_T	$E_{\text{T}}(r) = E_{\text{T0}} e^{-\beta_{\text{T}} r}$	$\beta_T = 1.0 \pm 0.1$ nm<br>$E_{T0} = 1000 \pm 100$ meV

Supplementary Table 2. Input Parameters for Kinetic Monte Carlo Simulations, note for the electronic coupling, N_P is taken to be 2 in the simulations.

r (nm)	λ (meV)	V_{100} (meV)	V_{111} (meV)	E_T (meV)	E_{T111} (meV)	E_{T100} (meV)	V_{B0} (mV)	σ_g (meV)	p_T (%)
2.21	23.5	0.345	0.033	109.7	24.4	16.6	0.550	54.0±0.1	3.0±0.1
2.21	23.5	0.212	0.033	109.7	24.4	16.6	0.550	54.0±0.1	4.0±0.1
1.58	44.4	0.473	0.075	206.0	45.7	31.1	0.708	72.2±0.1	0.7±0.1
1.37	58.3	0.666	0.105	254.1	56.4	38.4	0.286	77.9±0.2	0.3±0.1

Supplementary References

(1) Yazdani, N.; Bozyigit, D.; Vuttivorakulchai, K.; Luisier, M.; Infante, I.; Wood, V. Tuning Electron–Phonon Interactions in Nanocrystals through Surface Termination. *Nano Lett.* **2018**, *18* (4), 2233–2242. https://doi.org/10.1021/acs.nanolett.7b04729.

(2) Bixon, M.; Jortner, J. Electron Transfer—from Isolated Molecules to Biomolecules. In *Advances in Chemical Physics*; 1999; pp 35–202. https://doi.org/10.1002/9780470141656.ch3.

(3) Bozyigit, D.; Yazdani, N.; Yarema, M.; Yarema, O.; Lin, W. M. M.; Volk, S.; Vuttivorakulchai, K.; Luisier, M.; Juranyi, F.; Wood, V. Soft Surfaces of Nanomaterials Enable Strong Phonon Interactions. *Nature* **2016**, *531* (7596), 618–622. https://doi.org/10.1038/nature16977.

(4) Grinolds, D. D. W.; Brown, P. R.; Harris, D. K.; Bulovic, V.; Bawendi, M. G. Quantum-Dot Size and Thin-Film Dielectric Constant: Precision Measurement and Disparity with Simple Models. *Nano Lett.* **2015**, *15* (1), 21–26. https://doi.org/10.1021/nl5024244.

(5) Lin, S. H.; Chang, C. H.; Liang, K. K.; Chang, R.; Shiu, Y. J.; Zhang, J. M.; Yang, T.-S.; Hayashi, M.; Hsu, F. C. Ultrafast Dynamics and Spectroscopy of Bacterial Photosynthetic Reaction Centers. In *Advances in Chemical Physics*; John Wiley & Sons, Inc.: New York, USA, 2002; pp 1–88. https://doi.org/10.1002/0471264318.ch1.

(6) Reich, K. V; Shklovskii, B. I. Dielectric Constant and Charging Energy in Array of Touching Nanocrystals. *Appl. Phys. Lett.* **2016**, *108* (11), 113104. https://doi.org/10.1063/1.4944407.

(7) Bozyigit, D.; Lin, W. M. M. M.; Yazdani, N.; Yarema, O.; Wood, V. A Quantitative Model for Charge Carrier Transport, Trapping and Recombination in Nanocrystal-Based Solar Cells. *Nat. Commun.* **2015**, *6* (1), 6180. https://doi.org/10.1038/ncomms7180.
